# Development and implementation of multilocus sequence typing to study the diversity of the yeast *Kluyveromyces marxianus* in Italian cheeses

**DOI:** 10.1099/mgen.0.000153

**Published:** 2018-01-18

**Authors:** Fabrizia Tittarelli, Javier A. Varela, Loughlin Gethins, Catherine Stanton, R.P. Ross, Giovanna Suzzi, Luigi Grazia, Rosanna Tofalo, John P. Morrissey

**Affiliations:** ^1^​ Faculty of BioScience and Technology for Food, Agriculture and Environment, University of Teramo, Teramo, Italy; ^2^​ School of Microbiology, Environmental Research Institute, Centre for Synthetic Biology and Biotechnology, University College Cork, Cork T12YN60, Ireland; ^3^​ Teagasc Research Centre, Moorepark, Ireland; ^4^​ School of Microbiology, APC Microbiome Institute, University College Cork, Cork, Ireland; ^5^​ Department of Science and Technology for Food and Agriculture (DISTAL), Alma Mater Studiorum, University of Bologna, Bologna, Italy

**Keywords:** MLST, dairy products, *Kluyveromyces marxianus*, population

## Abstract

The yeast *Kluyveromyces marxianus* possesses advantageous traits like rapid growth, GRAS (generally regarded as safe) status and thermotolerance that make it very suitable for diverse biotechnological applications. Although physiological studies demonstrate wide phenotypic variation within the species, there is only limited information available on the genetic diversity of *K. marxianus*. The aim of this work was to develop a multilocus sequence typing (MLST) method for *K. marxianus* to improve strain classification and selection. Analysis of housekeeping genes in a number of sequenced strains led to the selection of five genes, *IPP1*, *TFC1*, *GPH1*, *GSY2* and *SGA1*, with sufficient polymorphic sites to allow MLST analysis. These loci were sequenced in an additional 76 strains and used to develop the MLST. This revealed wide diversity in the species and separation of the culture collection and wild strains into multiple distinct clades. Two subsets of strains that shared sources of origin were subjected to MLST and split decomposition analysis. The latter revealed evidence of recombination, indicating that this yeast undergoes mating in the wild. A public access web-based portal was established to allow expansion of the database and application of MLST to additional *K. marxianus* strains. This will aid understanding of the genetic diversity of the yeast and facilitate biotechnological exploitation.

## Data Summary

Accession numbers are provided in Table 1. The software used was as follows:

**Table 1.** *K. marxianus* strains used in this study and their origin

**Strain**	**Isolation source**	**Culture collection/accession no.**
LM3, LM6, LM9, LM14, LM15, LM17, LM20, LM28, LM30, LM32, LM36, LM38, LM42, LM44, LM47, LM48, LM50, LM54, LM72, LM92, LM96, LM114, LM116, LM127, LM133, LM136, LM139, LM141, LM142, LM148, LM153, LM154, LM161, LM167, LM169, LM174	Parmigiano Reggiano natural whey starter culture	Department of Agricultural and Food Sciences, University of Bologna, Italy [33]
6M2, 1SC4, K326	Parmigiano Reggiano cheese	
M12, M14, M38, M41, M48, M68, M81, M83, M123, M131, M135, M166, M169	Pecorino di Farindola cheese	Faculty of BioScience and Technology for Food, Agriculture, and Environment, University of Teramo, Italy [13]
VG1, VG4, VG6	Cow's milk whey	
FM09	Fermented milk	
CBS 4857, CBS 1555, CBS 5795, CBS 397, CBS 2762, CBS 6432, CBS 6556, CBS 712, CBS 7894, CBS 7858, CBS 1596, CBS 2233, CBS 4354, CBS 608, CBS 5668, CBS 745, CBS 834^T^		CBS-KNAW, Westerdijk Fungal Biodiversity Institute, Utrecht, The Netherlands
NCYC 179, NCYC 1424		National Collection of Yeast Cultures, UK
UCKM1	Unpasteurized cow's milk	School of Microbiology, University College Cork, Ireland [6]
DMKU 3-1042	Genome sequence	AP012220.1
NBRC 1777	Genome sequence	AP014606.1
CCT 7735	Genome sequence	CP009310.1
DMB1	Genome sequence	BBIL00000000.1
KCTC 17555	Genome sequence	AKFM00000000.2
IIPE453	Genome sequence	LDJA00000000.1
BO339	Genome sequence	LXZY00000000.1

Oligo Analyzer (https://eu.idtdna.com/calc/analyzer);ugene (http://ugene.net/);Mega 7 (http://megasoftware.net/);SnapGene (https://snapgene.com/);SplitsTree (http://splitstree.org/).

**Impact Statement**

*Kluyveromyces marxianus* is emerging as one of the most important non-*Saccharomyces* yeasts under development for biotechnological applications. It has traits such as rapid growth, high yield and thermotolerance that make it relevant for exploitation. In addition, genome engineering tools are now available to allow construction of new strains. There is, however, little information on the genetic diversity of the species, or on the relationship between strains. This is resolved in this study through the development of an multilocus sequence typing system. To facilitate use by the research community, a web-based database and tool was implemented to allow any researchers to easily type and classify more *K. marxianus* strains in the future.

## Introduction

*Kluyveromyces marxianus* is a homothallic yeast that, along with its sister species *Kluyveromyces lactis*, belongs to the subphylum Saccharomycotina. Some strains are able to utilize lactose as a carbon source but, unlike *K. lactis*, it can also secrete enzymes that degrade inulin, a fructan found in plants such as Jerusalem artichokes and agave [1, 2]. The capacity to assimilate lactose means that *K. marxianus* is frequently isolated from dairy products, such as fermented milk, yoghurt, kefir, koumiss and cheese, although it can also be isolated from fermenting fruit and plant material [3–7]. The broad metabolic capacity combined with a rapid growth rate, thermotolerance (up to 52 °C) and a high secretory capacity (e.g. secretion of lytic enzymes) have led to multiple applications in biotechnology; for example, production of bioingredients and aroma compounds, production of ethanol by fermentation, removal of lactose from food, and bioremediation [8, 9]. Because of its long and safe association with foods, *K. marxianus* is a GRAS (generally regarded as safe) organism in the USA and is on the European Food Safety Authority QPS (qualified presumption of safety) list.

There is not a unique *K. marxianus* strain that has been extensively characterized as a ‘model’ and those strains that have been studied display substantial physiological diversity [2, 10]. Although a challenge for detailed molecular and metabolic studies, this diversity is likely to be a very important enabler of a wide spectrum of biotechnological applications and, thus, can be considered positively. Nonetheless, full exploitation of this potential requires an improved understanding of the genetic diversity, and a means to identify and categorize strains. Sequence variation in the D1/D2 region of the 26S rRNA gene is universally accepted as the best way to distinguish yeast species from each other [11], but since this sequence is invariant within a species, this is not useful for intraspecific studies. Several researchers in recent years have applied molecular approaches to address this question. Differences at the intraspecific level in *K. marxianus* have been studied by restriction analysis of the non-transcribed spacer (NTS) region of the rRNA gene cluster, RAPD (random amplified polymorphic DNA) PCR fingerprinting and variability of the insertion of the long terminal repeat (LTR) retrotransposon Tkm1 [12–16]. Karyotyping of *K. marxianus* strains from different isolation sources and geographical origins suggest diverse chromosome numbers, although analysis of genomes of sequenced strains is consistent with a standard complement of eight chromosomes [17, 18]. This fits the emerging view that stable haploid and diploid *K. marxianus* strains occur in natural environments [19]. It is notable, however, that a wide haplotype diversity was observed through RFLPs of mitochondrial DNA [15, 20]. A rapid, easy to use, scalable method in yeast and fungi is microsatellite analysis. Microsatellites are short tandem DNA repeats that can be found in all genomes and are used as molecular markers able to distinguish strains. They have been applied for typing a variety of yeasts, such as *Saccharomyces uvarum* and *Saccharomyces cerevisiae*. These studies showed that the patterns are stable and that the method is discriminatory; thus, it can be used for population structure studies [21, 22].

Comparison of the relationships among *K. marxianus* strains is difficult and does not yield consistent patterns [23]. Furthermore, none of those methods is based on phylogeny, and so including new isolates and strains is problematic. These drawbacks can be overcome with the use of methods that are based on genomic sequence variation, especially multilocus sequence typing (MLST), which is based on analysing polymorphic sites in a number of conserved housekeeping genes within a species, permits easy comparison and sharing of the results among laboratories, and minimizes the possibility of errors resulting from local experimental conditions [24]. MLST has frequently been used to evaluate the genetic structure of different bacteria and yeasts [25, 26]. For example, this technique has been applied to study population structure and evolution of *S. cerevisiae* [27–29] and of the pathogenic yeast *Candida albicans* [30–32]. The aim of this work was to develop MLST for *K. marxianus* to enable strain identification and classification. As proof of principle, this method was applied to study the diversity of a collection of *K. marxianus* strains that had been isolated from two Italian cheese fermentations.

## Methods

### Origin of yeast strains

The 83 *K. marxianus* strains included in this study are listed in Table 1, which also lists the strains for which genome sequences were available for this study. Most of the isolates were from dairy products from different geographical areas in Italy, and were obtained from previous studies [13, 33]. In addition, 19 *K. marxianus* strains were from the Westerdijk Fungal Biodiversity Institute (Utrecht, The Netherlands) and from the National Collection of Yeast Cultures (Norwich, UK), and 1 was from the University College Cork collection. Yeasts were maintained as culture stocks in YPD (1 %, w/v, yeast extract, 2 %, w/v, peptone, 2 %, w/v, dextrose) medium containing 20 % (v/v) glycerol at −80 °C. All the strains were routinely grown in YPD medium overnight at 28 °C in shake flasks.

### Choice of loci and design of primers

Five housekeeping genes were retrieved from the *K. marxianus* genomes of seven sequenced strains (CCT 7735, DMB1, DMKU 3-1042, NBRC 1777, KCTC 17555, IIPE453, BO339), and a multiple sequence alignment was carried out to identify single nucleotide polymorphisms (SNPs). These genes, *IPP1*, *TFC1*, *GPH1*, *GSY2* and *SGA1*, showed good SNP distribution and were used in this study. Primers were designed with SnapGene software to hybridize to non-variable regions of each gene, thereby allowing amplification of the polymorphic region in any *K. marxianus* strain. The primer sequences and amplicon sizes are shown in Table 2. A BLAST (basic alignment search tools) search was used to check the specificity for *K. marxianus* of each primer set. Furthermore, the properties of each primer were verified by Oligo Analyzer 3.1. *K. lactis* CBS 683^T^ was used as a negative control to evaluate primer specificity by PCR.

**Table 2.** Characteristics of the five housekeeping genes and primer sequences

**Gene**	**Molecular function**	**Gene length (bp)**	**Primer (5′→3′)**	**Amplicon size (bp)**	**5′ (3′) position start**	**Size of sequenced fragment (bp)**	**No. of genotypes identified**	**No. of variable sites**		
								**Polymorphic nucleotide site**	**Non-synonymous polymorphic amino acid site**	**Synonymous polymorphic amino acid site**
*IPP1*	Inorganic diphosphatase activity	864	Fwd: ATCGGTGCCAAGAACACCTT	803	44 (807)	763	28	25	4	21
			Rev: TTGTCGATTGGCTCGTCTGG							
*TFC1*	RNA Pol III transcription factor activity	1857	Fwd: AAGGCCGATTTGGGTCAAAC	832	992 (1783)	791	37	44	19	25
			Rev: TCTGCGGACTCAGAGTTATGC							
*GPH1*	Glycogen phosphorylase activity	2712	Fwd: TGGAACACTGTGAAGCAGCA	902	1797 (2659)	862	30	34	8	26
			Rev: TTTCGTCAGCGTACTCCTGG							
*GSY2*	Glycogen synthase activity	2106	Fwd: CACGCCATGAGATTCCCTCA	891	1197 (2068)	871	34	49	19	30
			Rev: CGTCCTCTTCGTCGTCATCC							
*SGA1*	Glucan 1,4-α-glucosidase activity	1788	Fwd: CTCCGATGGTTCGGGTCAAT	926	179 (1085)	906	26	44	13	31
			Rev: TCATGGGTCAAGGTACTGGC							

### DNA extraction, amplification and nucleotide sequencing

DNA was extracted with the QIAprep Spin miniprep kit, following the manufacturer’s guidelines (Qiagen), modiﬁed as follows: 5 ml overnight culture was resuspended in 250 µl resuspension buffer [50 mM Tris-HCl (pH 8.0), 10 mM EDTA and 10 µg RNAse A ml^−1^) and vortexed for 5 min with glass beads (0.15–0.6 mm in diameter; Sigma). DNA quantification was carried out with a NanoDrop ND-1000 spectrophotometer. PCR analysis was performed with a ProFlex PCR system thermal cycler (Applied Biosystems). The reaction mixture (25 µl) contained 100 ng genomic DNA, 13 µl GoTaq Green master mix (Promega) and 10 µM each primer. The PCR conditions were: initial denaturation at 95 °C for 5 min; followed by 30 cycles of denaturation at 95 °C for 30 s, annealing at 56 °C for 15 s and elongation at 72 °C for 1 min; followed by a final extension step at 72 °C for 5 min. PCR products were separated in 1 % (w/v) agarose gel in 1× TAE buffer [40 mM Tris (pH 7.6), 20 mM acetic acid, 1 mM EDTA (pH 8.0)] at 80 V for 1 h, stained with SafeView (NBS Biologicals) and visualized under UV light. DNA fragment sizes were determined using a 1 kb DNA HyperLadder (BioLine). The amplified products were purified with a commercial PCR purification system (QIAquick PCR purification kit; Qiagen), and amplicons were sequenced in each direction by GATC (Constance, Germany).

### Data analysis

The sequence analysis for each gene fragment was performed with Mega 7 [34] and Unipro ugene programs [35]. For each fragment, the sequences obtained from the 83 strains were manually edited and compared with those from the 7 *K. marxianus* reference sequences. Defined regions in all sequences obtained from a single locus were aligned using Muscle [36]. All the polymorphic sites were visually inspected, and the polymorphic nucleotide sites within these sequences were identified. In some cases, there were two coincident, equivalently sized peaks in the forward and reverse sequences, indicating heterozygosity at that position. To avoid erroneous interpretation of double peaks, we followed previous suggestions, and routinely took account of noise in neighbouring positions and compared the chromatograms for both sequenced strands [37]. The one-letter code for nucleotides from the International Union of Pure and Applied Chemistry (IUPAC) nomenclature was used to designate homozygous and heterozygous polymorphic sites. Genotype numbers were assigned to each unique sequence. Each strain was defined by the combination of numbers corresponding to the genotype at the loci analysed, which is a genotype profile or diploid sequence type (DST). Sequences different even at a single nucleotide site were considered distinct genotypes.

Similarities among allelic profiles were displayed with a dendrogram generated by using the unweighted pair-group method with average (UPGMA) algorithm. This data analysis was performed by using start software [38]. For the computation of the phylogenetic tree, based on the concatenated sequence of the five loci, a distance matrix was created and a phylogenetic tree was reconstructed with the program Unipro ugene by using the maximum-likelihood method. The bootstrap analysis was conducted on 1000 replicates.

The MLST data were used to predict genetic exchanges with the program SplitsTree [39]. The split decomposition method was used to assess the degree of tree-like structure present in the alleles found in all the five loci for the strains isolated from the Italian cheeses. Three types of statistical analysis were applied to the data: (1) the *p*-distance to check mean identity to estimate reliability of the alignment; (2) the standardized index of association (I^S^_A_) to estimate multilocus linkage disequilibrium [40, 41]; (3) the *phi*-test for detecting the presence of recombination. The tests were carried out with Mega 7, start and SplitsTree programs, respectively.

### Website development

A website was created to allow users to compare a strain of interest against the 83 *K. marxianus* strains shown in this study, through a maximum-likelihood phylogenetic tree. The website takes a multi fasta file containing the *IPP1*, *TFC1*, *GPH1*, *GSY2* and *SGA1* sequences as input. Then, it runs a custom Biopython script that computes the phylogenetic tree. The program checks for sequence format, runs a multiple alignment against a prebuilt alignment profile using muscle v 3.8 and reconstructs the phylogenetic tree using PhyML v 3.1. The website was constructed using the HTML v 5.0 mark-up language and CSS v 3. Complementary scripts were written in Biopython v 1.69 and implemented into the website through a CGI Python script.

## Results and Discussion

The availability of an accurate and reproducible method to classify strains within a species is necessary to establish phylogenetic relationships and study biogeography, population structure and evolution. There are a wide variety of methods that have been developed and applied for this purpose but, depending on the method, there can be problems with technical difficulty, poor reproducibility between groups and limited scalability [42, 43]. The most powerful methods are based on DNA sequence variation; thus, comparison of whole genome sequences can be considered the gold standard. However, despite the reduction in sequence costs, this is not always feasible when large numbers of strains are involved and other methods that are rapid, easy to use and scalable, while still accurate, have been applied over the years to study diversity [16–18, 20].

### Choice of loci and sequence polymorphisms

The focus of this study was to develop an MLST analysis method for *K. marxianus* that could be used to distinguish strains. The utility of MLST relies on the selectively neutral variability of the housekeeping genes selected [24]; therefore, the choice of genes is critical.

To identify loci that would be suitable for MLST, five genes that had been previously described as housekeeping genes and used as controls in molecular studies in *S. cerevisiae* were considered [44]. The sequences of these genes were retrieved from the seven *K. marxianus* genome sequences available at that time. The dN/dS (non-synonymous to synonymous amino acid changes) in each of the five genes, *IPP1 (0)*, *TFC1* (0.03±0.013), *GPH1* (0.006±0.003), *GSY2* (0.04±0.017) and *SGA1* (0.04±0.036) was less than 1, indicating that they were not under positive selection; therefore, they were valid choices for MLST. These genes were individually compared and it was confirmed that they satisfied the criterion of displaying sufficient polymorphisms within regions that could be comfortably amplified in a single PCR reaction. Seventy-nine SNPs were found within the selected sequences of the five genes and primers to conserved regions were then designed to PCR amplify these loci from 76 additional *K. marxianus* strains. The identical loci were successfully amplified from all strains and analysed for polymorphisms. In total, there were 196 polymorphic sites (4.6 %) where nucleotide substitutions were seen. No insertions or deletions were seen and 133/196 of the changes were synonymous, not changing the predicted encoded amino acid. The remaining 63 SNPs resulted in a changed predicted amino acid at that position, of which 39 were substantive, such as basic to acid side chains and aliphatic to aromatic side chains (Table S1, available in the online version of this article). The most common change was from a non-polar aliphatic side chain to a polar aliphatic side chain. The number of polymorphic sites ranged from 25 in *IPP1* to 49 in *GSY2* (Table 2). Phylogenetically informative sites (at least two bases present in two or more of the alleles) ranged from 67 % in *TFC1* and *GPH1* to 41 % in *GSY2*, and are also shown in Table S2, which also details polymorphic nucleotide sites, the different genotypes identified at each of the five genes and their frequency of detection among the 83 *K. marxianus* strains.

All fragments of a given locus were aligned by multiple sequence alignment and a reference sequence was defined on the basis of the most common nucleotide at each polymorphic site (Table S2). Many of the strains were diploids and heterozygosity was quite common, in which case it was necessary to use IUPAC nomenclature to describe the sequence. It was not possible to determine which allele was associated with which nucleotide at heterozygous positions, a limitation common to all methods using nucleotide polymorphism analysis at several heterozygous loci in diploid organisms [30]. The reference sequence was given an arbitrary genotype number of 1, with variant sequences numbered successively (Table S2). The number of genotypes identified for each of the five loci investigated ranged from 26 for *SGA1* to 37 for *TFC1* (Table 2). Using the five genotype numbers, it was possible to describe the DSTs as the unique combination of the genotypes of the five loci studied. From the 83 strains, 66 DSTs were defined, whereby 58 strains were the sole representative of their DST, while the other 8 DSTs contained 2–7 representatives. This high level of differentiation confirmed the discriminatory power of MLST as a strain-typing procedure (Table 3).

**Table 3.** Genotypes of the 83 strains of *K. marxianus* analysed by MLST

**Strain name**	**Genotype**					**DST**
	***IPP1***	***TFC1***	***GPH1***	***GSY2***	***SGA1***	
LM127*, LM30*, LM38*	1	1	1	1	1	**1**
LM3*, LM50*, LM96*	1	1	1	1	14	**2**
LM47*	1	1	1	1	18	**3**
LM32*	1	1	1	2	1	**4**
LM6*	1	1	1	4	2	**5**
LM42*	1	1	1	17	16	**6**
LM36*	1	1	6	1	1	**7**
CBS 1555, CBS 712, CBS 7858, CBS 608	1	1	8	1	1	**8**
6M2*	1	1	10	3	4	**9**
CBS 834^T^	1	1	11	1	4	**10**
VG6†	1	1	16	17	2	**11**
K326*	1	1	26	16	4	**12**
M169‡, M38‡	1	1	26	17	4	**13**
LM139*	1	4	1	1	1	**14**
LM133*	1	4	1	1	15	**15**
CBS 7894	1	3	9	22	1	**16**
1SC4*	1	6	13	12	15	**17**
LM116*	1	7	1	7	15	**18**
LM72*	1	12	4	1	1	**19**
LM28*	1	19	5	28	1	**20**
LM169*	1	20	27	13	24	**21**
LM142*	1	21	7	1	25	**22**
CBS 2762	1	25	28	8	14	**23**
CBS 1596	1	32	21	1	1	**24**
LM141*	1	33	1	1	1	**25**
LM92*	1	33	8	20	1	**26**
LM174*	1	34	2	19	22	**27**
LM9*	1	35	1	1	14	**28**
LM14*	2	1	1	1	3	**29**
LM48*	3	1	1	5	1	**30**
LM136*	4	9	4	6	15	**31**
LM17*	5	15	1	10	23	**32**
LM114*	6	23	1	18	1	**33**
LM153*	7	17	3	11	21	**34**
VG1†, VG4†	8	11	22	22	11	**35**
M41‡	8	13	22	17	4	**36**
M83‡	8	14	22	17	4	**37**
M12‡, M123‡, M131‡, M135‡, M166‡, M48‡, M68‡	9	1	26	17	4	**38**
M14‡	9	1	26	17	5	**39**
M81‡	10	13	22	17	4	**40**
LM44*	11	1	1	1	15	**41**
CBS 397	12	1	8	1	1	**42**
LM161*	13	26	14	14	20	**43**
LM15*	13	27	12	15	17	**44**
LM154*	13	37	16	32	20	**45**
FM09§	14	31	29	29	15	**46**
NCYC 179	15	1	19	1	7	**47**
CBS 6432	15	1	24	1	7	**48**
UCKM1||	15	36	17	33	6	**49**
CCT 7735	16	22	26	30	15	**50**
NCYC 1424	16	28	14	9	14	**51**
CBS 5795	16	29	14	9	14	**52**
LM54*	17	12	4	1	1	**53**
CBS 5668	19	24	15	25	14	**54**
BO339	18	3	30	34	8	**55**
KCTC 17555^T^, CBS 6556	20	5	25	21	13	**56**
NBRC 1777	21	1	22	22	11	**57**
CBS 4857	21	10	18	23	9	**58**
DMKU 3-1042	22	11	22	22	10	**59**
LM148*	23	11	18	31	26	**60**
IIPE453	22	11	22	22	11	**61**
CBS 745	24	30	20	26	11	**62**
DMB1	25	11	18	22	12	**63**
LM20*	26	18	13	24	19	**64**
LM167*	27	16	18	27	11	**65**
CBS 2233, CBS 4354	28	8	23	23	11	**66**

*From Parmigiano Reggiano, Italy.

†From cow's milk whey, Italy.

‡From Pecorino di Farindola, Italy.

§From fermented milk, Italy.

||From unpasteurized milk, Ireland.

**Fig. 1.**
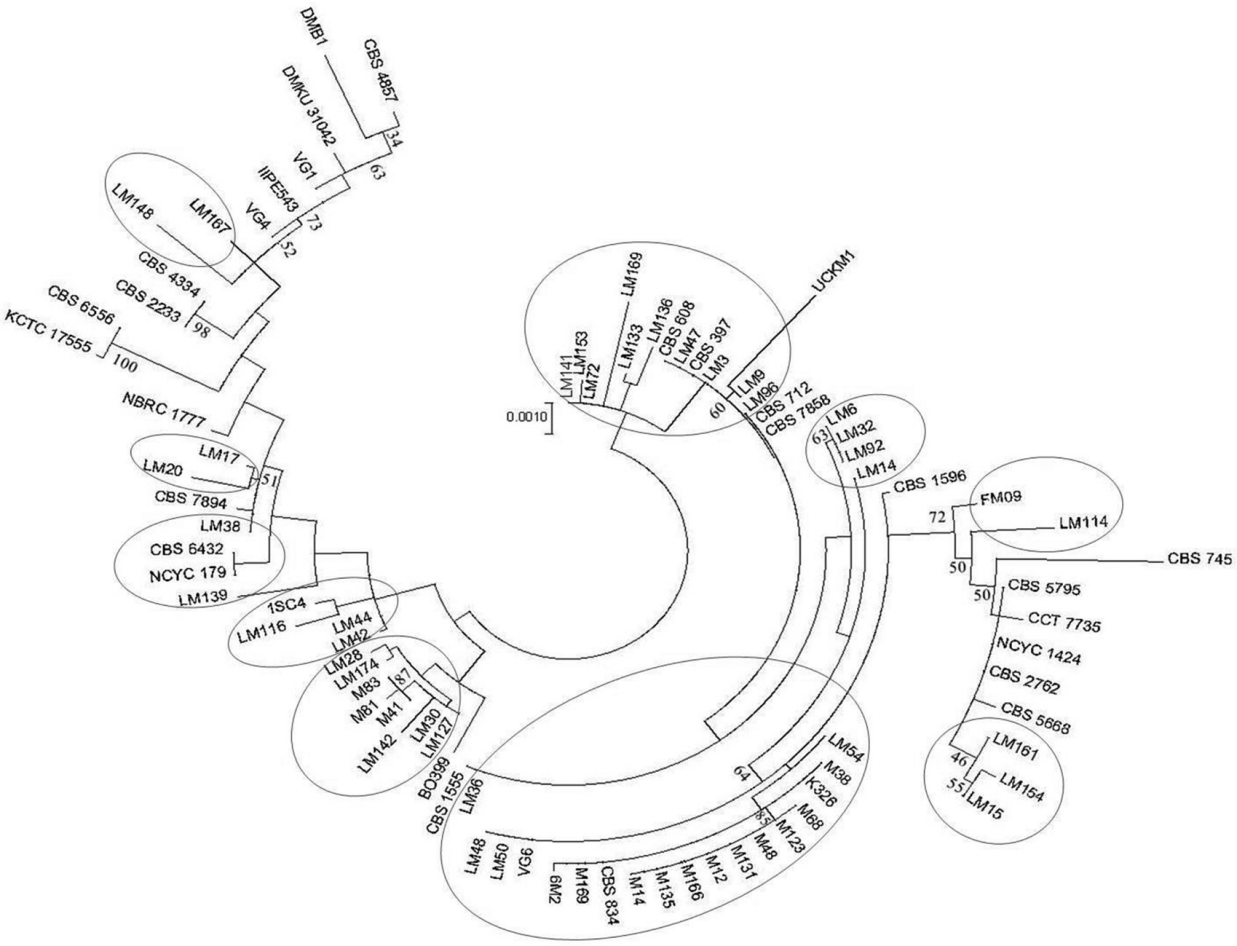
Phylogenetic tree reconstructed on the basis of the aligned concatenated sequence of the five housekeeping genes used in this work (*IPP*1, *TFC*1, *GPH*1, *GSY*2, *SGA1*) with the maximum-likelihood method. The position of the strains originating from the two Italian cheeses are indicated by circles. The bootstrap analysis was conducted on 1000 replicates.

**Fig. 2.**
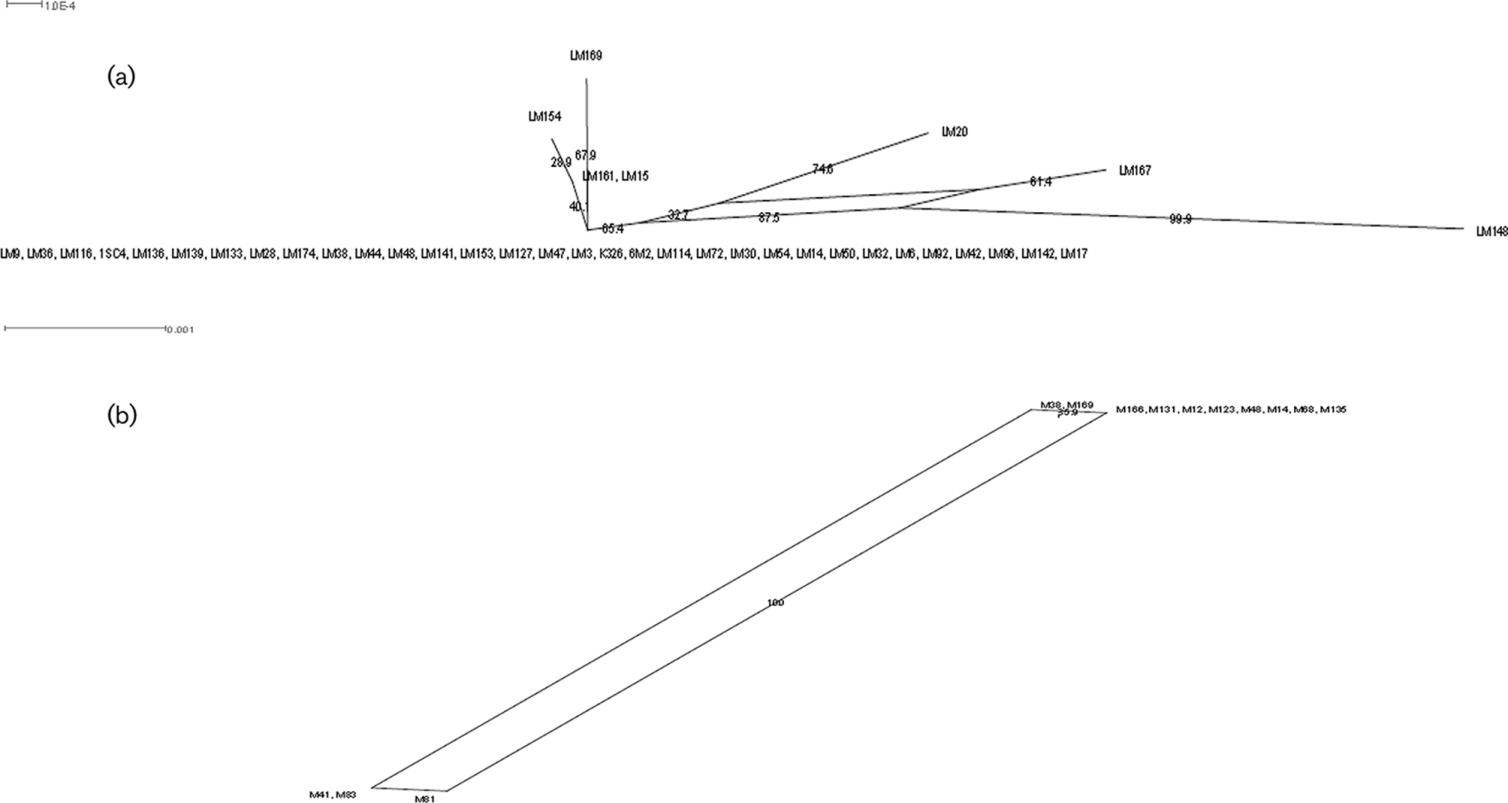
Split decomposition analysis based on the allelic profiles of the 39 strains isolated from Parmigiano Reggiano (a), and the 13 strains isolated from Pecorino di Farindola (b).

To investigate the relationship between the 83 strains, the 5 loci of each strain were concatenated and aligned using Muscle. Allelic profiles evaluated by the UPGMA algorithm are shown in Fig. S1. A phylogenetic tree was then reconstructed by the maximum-likelihood method using Unipro ugene (Fig. 1). This illustrated the diverse nature of the *K. marxianus* species, and showed that the culture collections and sequenced strains are interspersed in different clades. Split decomposition analysis was applied to further analyse the population structure of the 52 *K. marxianus* strains that were isolated from two different Italian cheeses, Parmigiano Reggiano and Pecorino di Farindola. These strains were part of sets previously analysed by different typing methods that did not yield consistency in classification [13, 18, 33]. The concatenated *IPP1*, *TFC1*, *GPH1*, *GSY2* and *SGA1* gene sequence was analysed and graphically displayed with SplitsTree, which calculates a phylogenetic network that permits the visualization of genetic exchanges in addition to mutations (Fig. 2). The SplitsTree graphs for both the Parmigiano Reggiano strains (Fig. 2a) and the Pecorino di Farindola strains (Fig. 2b) presented network-like structures, separating the strains into groups. The same groups are found in the maximum-likelihood tree shown in Fig. 1. Concerning the origin of the strains, all *K. marxianus* isolated from Pecorino di Farindola clustered together, although strains M41, M81 and M83 appeared in a separated branch in a similar way as in Figs 1 and 2(b). However, Parmigiano Reggiano *K. marxianus* strains were found to be more different, showing the presence of different groups in Fig. 1. In Fig. 2(a) the majority of the strains clustered together and just seven strains (LM154, LM169, LM161, LM15, LM20, LM167 and LM148) were differentiated. Two different analyses were employed to check for recombination in the two sets of strains. First, the *phi*-test gave *p*-values of 1.337^−5^ with *K*=2 (Parmigiano Reggiano strains) and 2.158^−5^ with *K*=1 (Pecorino di Farindola strains), which provides statistical support for recombination in both groups of strains. Second, the level of linkage disequilibrium between alleles was assessed using the I_A_ value [45], which is calculated as the ratio of the observed variance with the expected variance [40]. To avoid dependence on the number of loci, the I^S^_A_, which is expected to be zero when alleles are in linkage equilibrium (free recombination), was used [41]. The I^S^_A_ value was 0.993 for the entire population, indicating the presence of groups of strains corresponding to distinct populations. The I^S^_A_ was then calculated for the two sub-populations separately (Parmigiano Reggiano and Pecorino di Farindola strains). The values were 0208 and 0269 for Parmigiano Reggiano and Pecorino di Farindola strains, respectively, indicating recombination within the subpopulations. Both the *phi*-test and the linkage disequilibrium measure I^S^_A_ are consistent with the idea that there is recombination within these populations of strains. This should not be unexpected since most strains were diploids and *K. marxianus* possesses an intact mating system and can readily be crossed in laboratory studies [19, 46].

To allow the MLST developed here to be applied to more strains, a straightforward website was set up where custom Biopython scripts carry out the typing and generate a phylogenetic tree as well as a PhyML text output file in Newick format. That output file can be imported into other tree visualization software such as mega or TreeMe. This website is hosted on a University College Cork server at (kmarxianusMLST.ucc.ie). To type any additional *K. marxianus* strains, the *IPP1*, *TFC1*, *GPH1*, *GSY2* and *SGA1* genes need to be sequenced using the primers specified in Table 2. The sequences are then uploaded in a multi fasta file and the analysis is automatically performed. Illustrations of the outputs are available as supplementary data (Figs S2, S3 and S4).

In conclusion, in addition to shedding light on the population structure of the *K. marxianus* species, this study provides a framework useful to understanding genetic divergence. Our results showed that MLST of *K. marxianus* is highly reproducible and discriminatory, and superior to other methods used to date to classify strains. To enhance the applicability of the method, a website was constructed to allow typing and comparison of additional *K. marxianus* strains in the future.

## Data bibliography

1. The accession numbers of the genome sequences are listed in Table 1.

## Supplementary Data

1100 Supplementary File 1
