# Supplementary material for: Development and implementation of multilocus sequence typing to study the diversity of the yeast Kluyveromyces marxianus in Italian cheeses
Source: Microb Genom. 2018 Jan 18;4(2):e000153. doi: 10.1099/mgen.0.000153 (PMC5857380; doi:10.1099/mgen.0.000153)
Supplement: Supplementary File 1 [file mgen-4-153-s001.pdf]

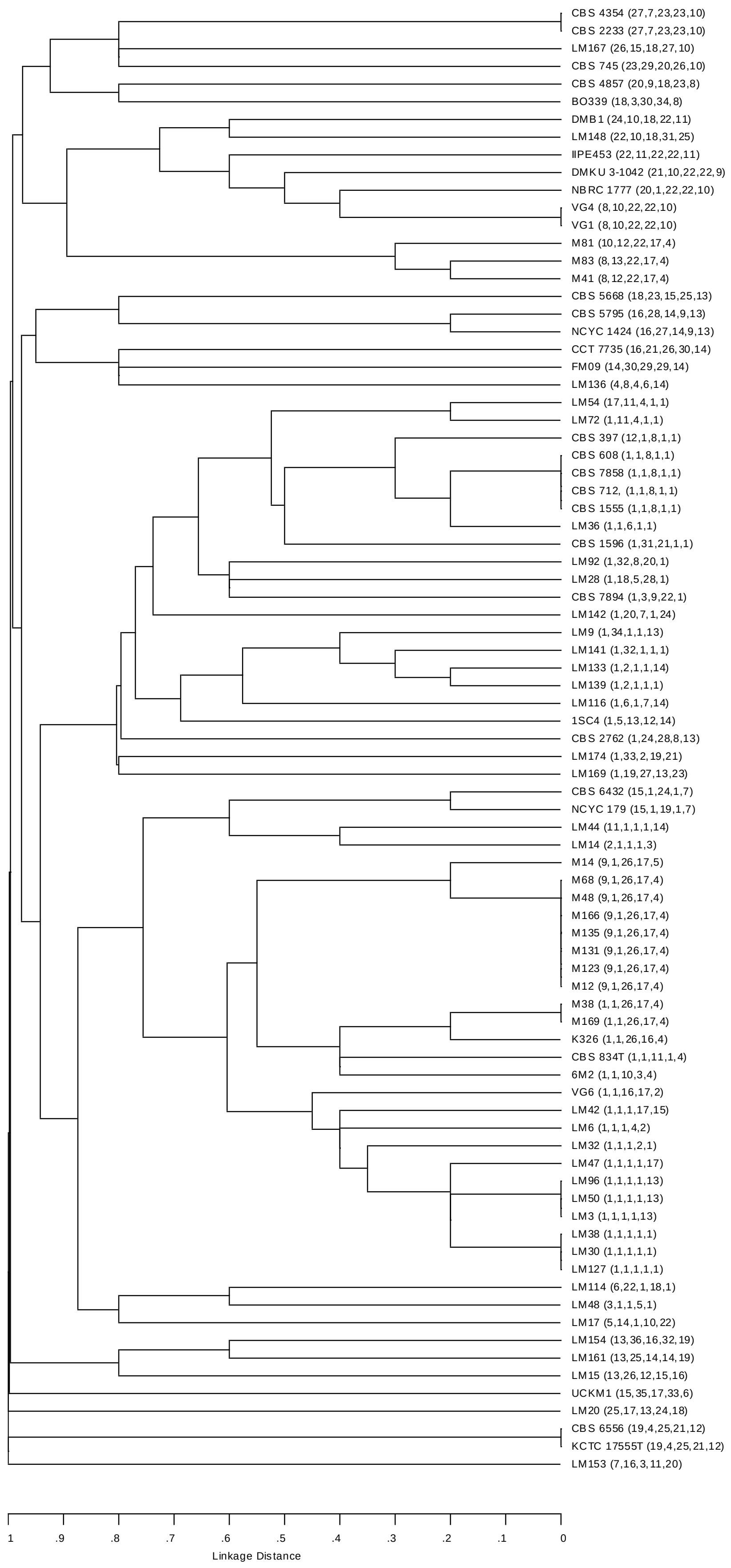

Figure S1. Allelic profiles evaluated by Unweighted Pair-Group Method with Average (UPGMA)

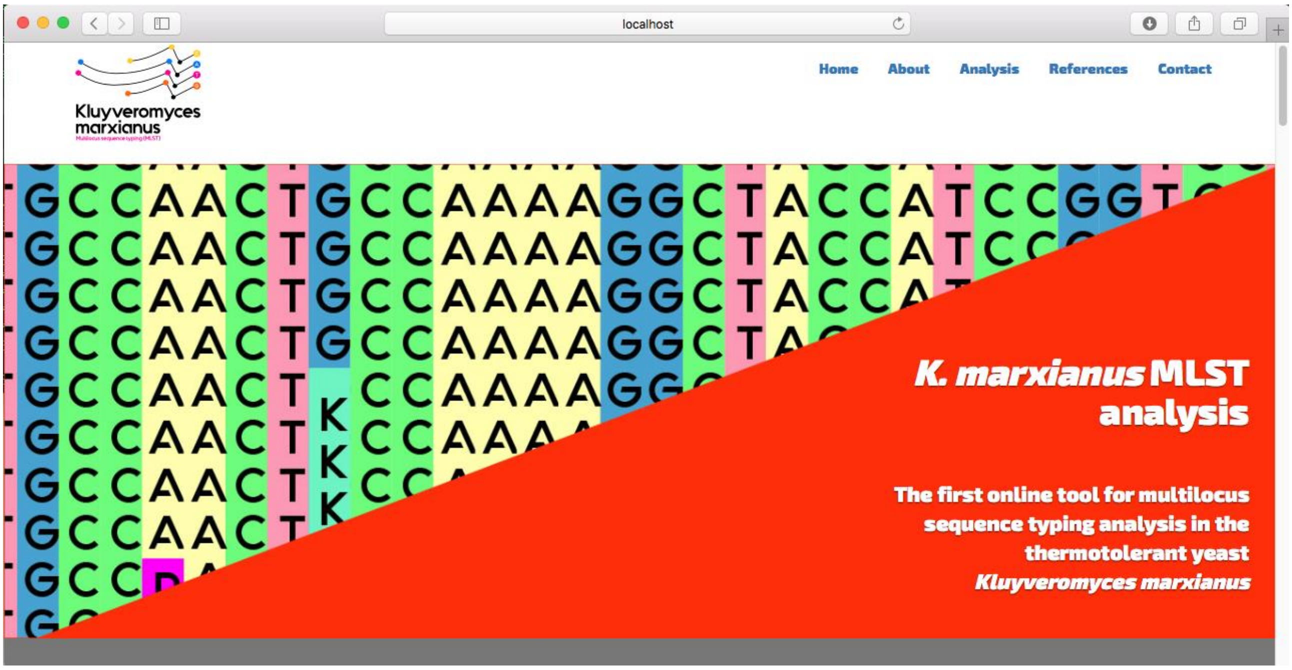

Figure S2. *K. marxianus* MLST analysis website home page

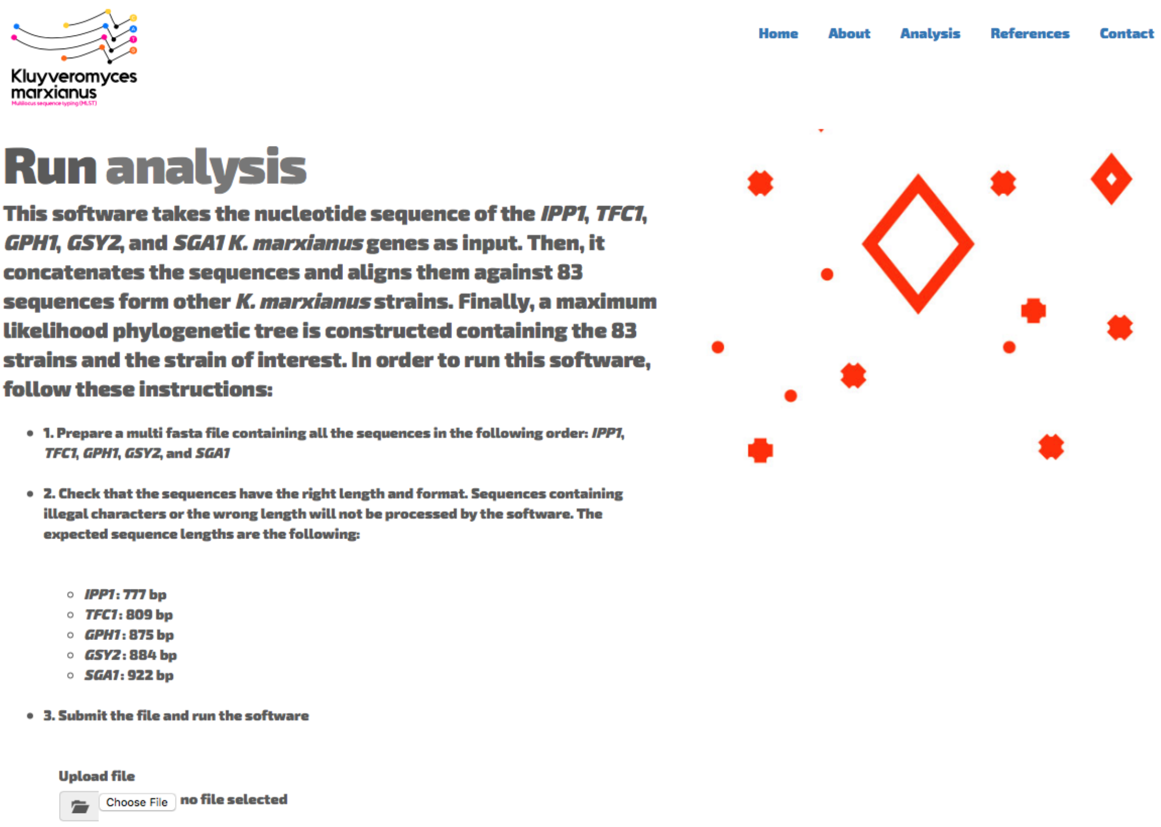

Figure S3. *K. marxianus* MLST analysis website run analysis page

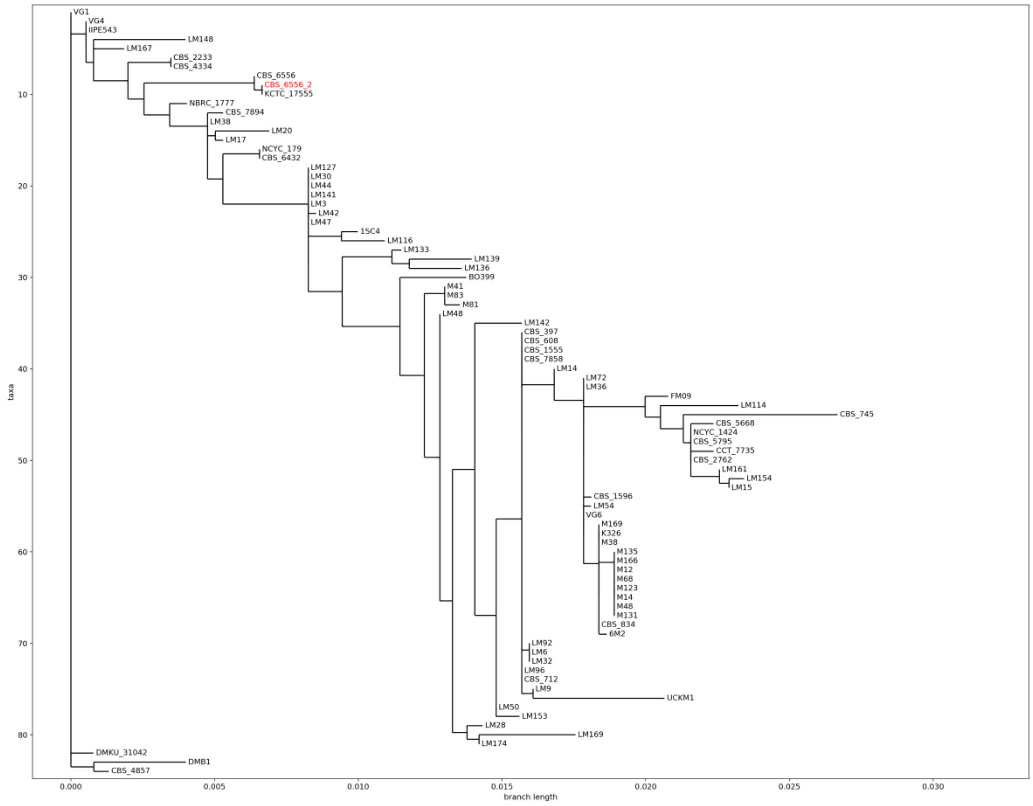

Figure S4. Output tree from *K. marxianus* MLST analysis website

Fragment	Starting nucleotide position of triplet	Consensus codon	Consensus amino acids	Triplet polymorphisms	Amino acid polymorphism
IPP1	40	AAG	Lys	AGC (9)	Ser
	637	AAC	Asn	CAC (1)	His
	691	GAW	Asp/Glu	GAT (20) - GAA (6)	Asp - Glu
	721	GCC	Ala	RCC (1)	Thr
TFC1	37	TTA	Leu	TTC (3)	Phe
	169	TAT	Tyr	TTT (1)	Phe
	178	ATC	Ile	ATG (1)	Met
	226	ACT	Thr	GCT (27) - RCT (12)	Ala - Ala/Thr
	259	CCT	Pro	MCT (1) - YCT (1)	Thr/Pro - Ser/Pro
	274	CCC	Pro	MCC (5)	Pro/Thr
	292	GGT	Gly	RGT (3)	Gly/Ser
	457	GAA	Glu	RAA (3)	Glu/Lys
	526	ATC	Ile	AYC (1)	Ile/Thr
	565	GAT	Asp	GAA (1)	Glu
	580	ATG	Met	TTG (1)	Leu
	592	AAC	Asn	AAG (1)	Lys
	595	GAC	Asp	GAG (1)	Glu
	643	GAG	Glu	GAC (2) - GAS (1)	Asp - Glu/Asp
	715	GAT	Asp	GAG (1)	Glu
	724	CAA	Gln	CGA (1)	Arg
	727	GAT	Asp	RAT (2)	Asp/Asn
	745	GAT	Asp	GAG (1)	Glu
	748	ACT	Thr	AST (3) - ACG (1)	Thr/Ser - Thr
GPH1	46	GAM	Asp/Glu	GAC (23) - GAA (23) - GAR (7)	Asp - Glu - Glu
	250	GGT	Gly	AAT (1)	Asn
	472	TTT	Phe	GTT (1)	Val
	511	GGT	Gly	KGT (1)	Gly/Cys
	544	GAC	Glu	GAK (1)	Glu/Asp
	769	TCS	Ser	TCC (24) - TCG (19) - TGC (1)	Ser - Ser - Cys
	775	GTC	Val	ATC (1)	Ile
	847	TTC	Phe	TTM (1)	Phe/Leu
GSY2	19	ACA	Thr	AGA (2)	Arg
	40	GAA	Glu	GGA (1)	Gly
	97	CGT	Arg	CCT (1)	Pro
	103	CCA	Pro	CMA (1)	Pro/Gln
	115	CTA	Leu	MTA (1) - CWA (1)	Leu/Ile - Leu/Gln
	118	CCC	Pro	CMM (1)	Pro/Gln/His
	151	GCT	Ala	GTT (1)	Ala/Val
	181	CGT	Arg	CRT (1)	Arg/His
	211	CGT	Arg	YGT (5)	Arg/Cys
	256	CCA	Pro	MMA (1)	Pro/Gln/Thr/Asn
	364	GGT	Gly	KGT (1)	Gly/Cys
	442	GAT	Asp	RAT (3)	Asp/Asn
	511	TAC	Tyr	WAC (1)	Tyr/Asn
	703	AAC	Asn	AGC (1)	Ser
	718	GCA	Ala	GGC (1)	Gly
	733	AAA	Lys	ASA (1)	Thr/Arg
	736	RTC	Val/Ile	GTC (32) - ATC (16)	Val - Ile
	817	CCC	Pro	YCC (1)	Pro/Ser
	856	GAT	Asp	AAT (3) - GAG (7) - GAY (1)	Asn - Glu - Asp
SGA1	40	GAC	Asp	GAA (2)	Glu
	43	TTT	Phe	CTT (2)	Leu
	46	CAA	Gln	TCA (2)	Ser
	97	CAA	Gln	MAA (2)	Gln/His
	112	CAG	Gln	MAG (3)	Gln/His
	349	ATC	Ile	RTC (3)	Ile/Val
	436	AAT	Asn	AGT (1) - ART (1)	Ser - Asn/Ser
	634	GAT	Asp	AAT (1) - RAT (2)	Asp - Asp/Asn
	742	RCA	Ala/Thr	GCA (20) - ACA (22)	Ala - Thr
	775	SAT	His/Asp	CAT (24) - GAT (17) - AAT (1)	His - Asp - Asn
	793	GAT	Asp	GAA (1)	Glu
	799	GTG	Val	GGG (1)	Gly
	907	GTC	Val	TTC (1)	Phe

Table S1. Changes in amino acid sequences resulting from nucleotide polymorphisms. The substantive changes are highlighted in grey. The numbers in bracket are the numbers of strains with the polymorphisms.

Table S2. Position of the polymorphic nucleotide sites and genotypes identified at loci *IPP1*, *TFC1*, *GPH1*, *GSY2*, *SGA1*. The most common sequence among the 83 *K. marxianus* tested is shown for genotype 1. For the other genotypes, only sites that differ from those in genotypes 1 are shown; sites that are the same as those in genotype 1 are shown by dots. The strains with the same genotype are indicated at the bottom of each table. The position of each polymorphic site relative to the fragment sequenced is shown at the top of the table. Asterisks indicate phylogenetically informative polymorphic sites. Y, C or T ; R, A or G; K, G or T; M, A or C, S, G or C; W, A or T.

IPP1 — Site position

Genotype	41	42	57	66	81	93	111	189	195	225	237	255	276	348	357	402	465	543	637	693	708	721	738	741	747
1	A	G	C	Y	C	Y	C	G	Y	A	C	C	C	G	A	C	C	C	A	W	K	G	A	T	W
2	.	.	.	.	.	.	.	S	.	.	.	.	.	.	.	.	.	.	.	.	.	.	.	.	.
3	.	.	.	.	.	.	.	.	.	.	.	.	.	.	.	.	.	S	.	.	.	.	.	.	.
4	.	.	.	T	.	.	.	.	.	.	.	.	.	.	.	.	.	.	.	.	.	.	.	.	.
5	.	.	.	.	T	T	.	.	.	.	.	.	.	.	.	.	.	.	.	.	.	.	.	.	.
6	.	.	.	.	.	T	.	.	.	.	.	.	.	.	.	.	.	.	.	.	.	R	.	.	.
7	.	.	.	.	.	.	.	.	.	.	.	Y	.	.	.	Y	.	.	.	.	G	.	.	.	.
8	.	.	.	.	.	.	.	.	.	.	.	Y	.	R	.	Y	Y	.	.	.	.	.	.	.	.
9	G	C	.	.	.	.	.	.	.	.	.	.	.	.	.	.	.	.	.	.	.	.	.	.	.
10	G	C	.	.	.	.	.	.	.	.	.	Y	.	R	.	Y	Y	.	.	.	.	.	.	.	.
11	.	.	.	.	.	T	.	.	.	.	.	.	.	.	.	.	.	.	.	T	G	.	.	.	T
12	.	.	.	T	.	C	.	.	C	.	.	.	.	.	.	.	.	.	.	.	.	.	.	.	A
13	.	.	.	T	.	C	.	.	C	.	.	.	.	.	.	.	.	.	.	T	T	.	.	.	.
14	.	.	.	T	.	C	.	.	C	.	.	.	.	.	.	.	.	.	.	A	T	.	.	.	.
15	.	.	.	T	.	C	.	.	C	.	.	.	.	.	.	.	.	.	.	A	T	.	.	.	A
16	.	.	.	T	.	C	.	.	C	.	.	.	.	.	.	.	.	.	.	T	T	.	.	.	A
17	.	.	.	C	.	C	.	.	C	.	.	.	.	.	.	.	.	.	.	A	T	.	.	.	A
18	.	.	.	C	.	C	.	.	C	.	.	.	.	.	.	.	.	.	.	T	G	.	.	.	A
19	.	.	.	T	.	C	.	.	C	.	.	.	.	.	.	.	.	.	C	T	T	.	.	.	A
20	.	.	.	C	.	C	.	.	T	.	.	T	.	A	.	.	.	.	.	T	G	.	.	.	T
21	.	.	.	C	.	T	.	.	T	.	.	T	T	A	.	T	.	.	.	T	G	.	.	.	T
22	.	.	.	C	.	T	.	.	T	.	.	T	.	A	.	T	T	.	.	T	G	.	.	.	T
23	.	.	.	C	.	T	.	.	T	.	.	T	.	A	.	T	T	.	.	T	G	.	.	.	.
24	.	.	.	T	.	C	.	.	C	W	.	.	.	.	R	.	.	.	.	A	T	.	.	.	G
25	.	.	.	C	.	T	.	.	T	.	.	.	T	A	.	.	.	.	.	T	G	.	G	A	T
26	.	.	T	C	.	T	.	.	T	.	T	.	.	.	.	.	.	.	.	T	G	.	.	.	.
27	.	.	.	T	.	T	.	.	.	.	.	T	.	R	.	Y	Y	.	.	.	.	.	.	.	.
28				C		T	S		C			Y				Y				T	G				T

Genotype 1: CBS 1596, CBS 7894, CBS 712, CBS 608, CBS 2762, CBS 7858, CBS 1555, CBS 834, LM50, LM6, LM72, LM9, LM92, LM96, LM47, LM30, LM32, LM36, LM42, LM3, LM141, LM38, LM127, LM142, LM174, LM169, LM133, LM116, LM139, M169, M38, K326, 6M2, 1SC4, VG6, LM28; **Genotype 2**: LM14; **Genotype 3**: LM48; **Genotype 4**: LM136; **Genotype 5**: LM17; **Genotype 6**: LM114; **Genotype 7**: LM153; **Genotype 8**: M41, M83, VG1, VG4; **Genotype 9**: M12, M14, M48, M68, M123, M131, M135, M166; **Genotype 10**: M81; **Genotype 11**: LM44; **Genotype 12**: CBS 397; **Genotype 13**: LM154, LM15, LM161; **Genotype 14**: FM09; **Genotype 15**: NCYC 179, CBS 6432, UCKM1; **Genotype 16**: NCYC 1424, CBS 5795, CCT 7735; **Genotype 17**:LM54; **Genotype 18**: BO339; **Genotype 19**:CBS 5668; **Genotype 20**: KCTC 17555, CBS 6556; **Genotype21**: CBS 4857, NBRC 1777; **Genotype 22**: DMKU 3-1042, IIPE453; **Genotype 23**: LM148; **Genotype 24**: CBS 745; **Genotype 25**: DMB1; **Genotype 26**: LM20; **Genotype 27**: LM167; **Genotype 28**: CBS 2233, CBS 4354

Continued on following page

Table S2 - *Continued*

*TFC*1 — Site position

Genotype	39	51	66	153	170	180	226	231	243	246	259	274	292	363	405	417	457	480	507	527	534	558	564	567	570	580	594	597	630	633	645	684	696	717	723	725	727	747	749	750	764	765	783	798
1	A	A	C	G	A	C	A	G	C	C	C	C	G	A	G	C	G	A	A	T	T	G	G	T	C	A	C	C	C	C	G	T	A	T	G	A	G	T	C	T	C	T	T	C
2	.	.	.	.	.	.	.	.	.	.	.	.	.	.	.	.	.	.	.	.	.	.	.	.	.	.	.	.	.	.	.	.	.	.	.	.	.	.	S	.	.	.	.	.
3	.	.	.	.	.	.	G	.	.	.	.	.	.	.	.	.	.	.	.	.	.	.	.	.	.	.	.	.	.	.	.	.	.	.	.	.	.	.	.	.	.	.	.	.
4	.	.	.	.	.	.	.	.	.	.	.	.	.	.	.	.	.	.	.	.	.	.	.	.	.	.	.	.	.	.	.	.	.	.	.	.	.	.	.	.	.	A	.	G
5	.	.	.	.	.	.	G	.	.	.	.	.	.	.	.	.	.	T	.	.	C	.	.	.	A	.	.	.	.	.	.	C	.	.	.	.	.	.	.	.	.	.	C	.
6	.	.	.	.	.	.	G	K	Y	Y	M	.	.	.	.	.	.	.	R	.	C	.	R	.	S	.	.	.	.	.	.	C	.	.	.	.	.	.	.	.	.	S	C	.
7	.	.	.	.	.	.	G	K	Y	Y	Y	.	.	.	.	.	.	.	R	.	C	.	R	.	S	.	.	.	Y	.	.	C	.	.	R	.	.	.	.	.	.	.	.	.
8	.	.	.	.	.	.	G	T	.	.	.	.	.	.	.	.	.	.	G	.	C	.	.	.	.	.	.	.	.	.	.	C	.	.	.	.	.	.	.	.	.	.	C	.
9	.	.	.	.	.	.	G	T	.	.	.	.	.	.	.	.	.	.	R	.	Y	.	.	.	.	.	.	.	.	.	.	C	.	.	.	.	.	.	.	.	.	A	.	G
10	.	.	.	.	.	.	G	T	.	.	.	.	.	.	A	.	.	.	G	.	C	.	.	.	G	.	.	.	.	.	.	C	.	.	.	.	.	.	.	.	.	.	.	.
11	.	.	.	.	.	.	G	T	.	.	.	.	.	.	A	.	.	.	G	.	C	A	.	.	G	.	.	.	.	.	.	C	.	.	.	.	.	.	.	.	.	.	.	.
12	.	.	.	.	.	.	R	K	.	.	.	.	.	.	.	.	.	.	R	.	Y	.	.	.	.	.	.	.	.	.	.	Y	.	.	.	.	.	.	.	.	.	.	Y	.
13	.	.	.	.	.	.	R	K	.	.	.	.	.	.	.	.	.	.	R	.	Y	A	R	.	S	.	.	.	.	.	.	Y	.	.	.	.	.	.	.	.	.	.	.	.
14	.	.	.	.	.	.	R	K	.	.	.	.	.	.	R	.	.	.	R	.	Y	A	R	.	S	.	.	.	.	.	.	Y	.	.	.	.	.	.	.	.	.	.	.	.
15	.	.	.	G	.	.	.	.	.	.	.	M	R	.	.	.	.	.	.	.	.	.	.	.	.	.	.	.	.	.	.	.	.	.	.	.	.	.	.	.	.	.	.	.
16	.	.	.	G	.	.	G	T	.	.	.	.	.	.	A	.	.	.	G	.	C	A	.	.	G	.	.	.	.	.	.	C	.	.	.	.	.	.	.	.	A	C	.	.
17	.	.	.	G	.	.	R	K	.	.	.	M	.	.	.	.	R	.	R	.	Y	.	.	.	.	.	.	.	.	.	.	Y	.	.	.	.	.	.	.	.	.	.	Y	.
18	.	.	.	G	.	.	R	K	.	.	.	M	R	.	R	.	R	.	R	.	Y	R	.	.	S	.	.	.	.	.	.	Y	G	.	.	G	.	G	.	G	.	.	.	.
19	.	.	.	G	T	G	.	.	.	.	.	.	.	.	.	.	.	.	.	.	.	.	.	.	.	.	.	.	.	.	.	.	.	.	.	.	.	.	.	.	.	.	.	.
20	.	.	R	G	.	.	.	T	.	.	.	.	R	.	.	.	.	.	.	.	.	.	.	A	.	T	G	G	.	.	.	.	.	.	.	.	.	.	.	.	.	.	.	.
21	.	G	.	.	.	.	R	.	Y	Y	.	M	.	.	.	.	.	.	.	.	Y	.	R	.	.	.	.	.	Y	.	.	.	.	.	.	.	.	.	.	.	.	W	Y	.
22	.	G	.	A	.	.	G	.	T	T	.	.	.	.	.	.	.	.	.	.	C	.	.	.	G	.	.	.	T	.	.	C	.	.	A	.	.	.	.	.	.	A	.	G
23	.	G	.	A	.	.	G	.	T	T	.	.	.	.	.	.	.	.	.	.	C	.	A	.	G	.	.	.	T	.	.	C	.	.	A	.	.	.	.	.	.	.	.	.
24	.	G	.	A	.	.	G	.	T	T	.	.	.	.	.	.	.	.	.	.	C	.	A	.	G	.	.	.	T	.	.	C	.	.	A	.	.	.	.	.	.	A	.	G
25	.	G	.	A	.	.	G	.	T	T	.	.	.	.	.	.	.	.	.	.	C	.	R	.	G	.	.	.	T	.	S	C	.	.	A	.	.	.	.	.	.	A	.	G
26	.	G	.	A	.	.	G	.	T	T	.	.	.	.	.	T	.	.	.	.	C	.	.	.	G	.	.	.	T	T	.	C	.	.	R	.	.	.	.	.	.	A	.	G
27	.	G	.	A	.	.	G	.	T	T	.	.	.	.	.	Y	.	.	.	.	C	.	R	.	G	.	.	.	T	Y	.	C	.	.	A	.	.	.	.	.	.	A	.	.
28	.	G	.	A	.	.	G	.	T	T	.	.	.	.	.	Y	.	.	.	.	C	.	R	.	G	.	.	.	T	Y	.	C	.	.	A	.	R	.	.	.	.	A	.	G
29	.	G	.	A	.	.	G	.	T	T	.	.	.	.	.	Y	.	.	.	.	C	.	R	.	G	.	.	.	T	Y	.	C	.	.	A	.	R	.	S	.	.	A	.	G
30	.	G	.	A	.	.	G	.	T	T	.	.	.	T	.	.	.	.	.	.	C	.	.	.	G	.	.	.	T	.	C	C	.	G	A	.	.	.	.	.	.	A	.	G
31	.	G	.	A	.	.	G	G	T	T	.	.	.	.	.	.	.	.	.	.	C	.	.	.	G	.	.	.	T	.	C	.	.	.	.	.	.	.	.	.	.	.	.	.
32	.	R	.	.	.	.	R	.	.	.	.	.	.	.	.	.	.	.	.	.	Y	.	R	.	S	.	.	.	Y	.	.	Y	.	.	R	.	.	.	S	.	.	W	.	S
33	.	R	.	R	.	.	R	.	Y	Y	.	.	.	.	.	.	.	.	.	.	Y	.	R	.	S	.	.	.	Y	.	.	Y	.	.	R	.	.	.	.	.	.	W	.	S
34	.	R	R	R	.	.	R	.	Y	Y	.	.	.	.	.	.	.	.	.	Y	Y	.	R	.	S	.	.	.	.	.	.	Y	.	.	.	.	.	.	.	.	.	.	Y	.
35	C	.	.	.	.	.	.	.	.	.	.	.	.	.	.	.	.	.	.	.	.	.	.	.	.	.	.	.	.	.	.	.	.	.	.	.	.	.	.	.	.	.	.	.
36	C	G	.	.	.	.	.	.	.	.	.	.	.	.	.	.	.	.	.	.	.	.	.	.	.	.	.	.	.	.	.	.	.	.	.	.	.	.	.	.	.	.	.	.
37	C	G	.	A	.	.	G	.	T	T	.	M	.	.	.	Y	R	.	.	.	C	.	R	.	G	.	.	.	T	Y	.	C	.	.	A	.	.	.	.	.	.	A	.	.
	*	*	*	*			*	*	*	*	*	*	*		*	*	*		*		*	*	*		*				*	*	*	*			*		*		*			*	*	*

Genotype 1: LM127, CBS 397, CBS 712, CBS 608, NCYC 179, CBS 6432, CBS 7858, CBS 1555, LM44, LM50, LM6, LM96, VG6, LM3, LM38, LM42, LM47, LM48, LM30, LM32, LM36, NBRC 1777, CBS 834, 6M2, K326, LM14, M12, M123, M131, M135, M14, M166, M169, M38, M48, M68; **Genotype 2**: CBS 7894; **Genotype 3**: BO339; **Genotype 4**: LM133, LM139; **Genotype 5**: CBS 6556, KCTC 17555; **Genotype 6**: 1SC4; **Genotype 7**: LM116; **Genotype 8**: CBS 4354, CBS 2233; **Genotype 9**: LM136; **Genotype 10**: CBS 4857; **Genotype 11**: VG1, LM148, DBM1, DMKU 3-1042, VG4, IIPE453; **Genotype 12**: LM72, LM54; **Genotype 13**: M41, M81; **Genotype 14**: M83; **Genotype 15**: LM17; **Genotype 16**: LM167; **Genotype 17**: LM153; **Genotype 18**: LM20; **Genotype 19**: LM28; **Genotype 20**: LM169; **Genotype 21**: LM142; **Genotype 22**: CCT 7735; **Genotype 23**: LM114; **Genotype 24**: CBS 5668; **Genotype 25**: CBS 2762; **Genotype 26**: LM161; **Genotype 27**: LM15; **Genotype 28**: NCYC 1424; **Genotype 29**: CBS 5795; **Genotype 30**: CBS 745; **Genotype 31**: FM09; **Genotype 32**: CBS 1596; **Genotype 33**: LM92, LM141; **Genotype 34**: LM174; **Genotype 35**: LM9; **Genotype 36**: UCKM1; **Genotype 37**: LM154

Continued on following page

Table S2 – *Continued*

*GPH*1 Genotype	Site position 6	24	48	72	150	153	183	204	213	250	251	264	324	348	354	366	369	426	459	472	511	522	546	594	666	705	726	732	770	771	775	792	849	855
1	C	C	M	C	R	Y	G	Y	G	G	G	R	C	Y	R	Y	W	Y	M	T	G	Y	G	R	Y	Y	Y	R	C	S	G	Y	C	C
2	.	.	.	.	.	.	.	.	.	.	.	.	.	.	.	.	.	.	A	.	.	.	.	.	.	.	T	.	.	.	.	.	.	M
3	.	.	.	.	.	.	.	.	.	.	.	.	.	.	.	.	.	C	C	.	.	.	K	.	.	.	T	.	.	.	.	C	.	.
4	.	.	.	.	.	.	.	.	.	.	.	.	.	.	.	C	T	C	C	.	.	.	.	.	.	.	.	.	.	.	.	.	.	.
5	.	.	.	.	.	.	.	.	.	.	.	.	.	.	.	T	.	.	.	.	K	.	K	.	.	.	.	.	.	.	.	.	.	.
6	.	.	.	.	.	T	.	.	.	.	.	.	.	.	.	.	.	.	.	.	.	.	.	.	.	.	.	.	.	.	.	.	.	.
7	.	.	C	.	.	.	.	.	.	.	.	.	.	.	.	.	.	.	.	.	.	.	.	.	.	.	.	.	.	.	.	.	.	.
8	.	.	R	.	.	.	.	.	.	.	.	.	.	.	.	.	.	.	.	.	.	C	.	.	.	.	.	.	.	.	.	.	.	.
9	.	.	R	.	.	.	.	.	.	.	.	.	.	.	.	.	.	.	.	G	.	C	.	.	.	.	.	.	.	.	.	.	.	.
10	T	T	.	.	.	.	.	.	.	.	.	.	.	.	.	.	.	.	.	.	.	.	.	.	.	.	.	.	.	.	.	.	.	.
11	T	T	.	.	.	.	.	.	.	.	.	.	.	.	.	.	.	.	.	.	.	.	.	.	.	.	C	.	.	C	.	.	.	.
12	.	.	.	.	A	T	.	C	.	.	.	G	.	.	A	C	T	C	C	.	.	T	.	A	T	C	C	G	.	C	.	T	.	.
13	.	.	C	.	G	C	.	T	.	.	.	A	.	C	G	T	A	.	A	.	.	C	.	G	C	T	T	A	.	G	.	C	.	.
14	.	.	A	.	A	T	.	C	.	.	.	G	.	.	A	C	T	C	C	.	.	T	.	A	T	C	C	G	.	C	.	T	.	.
15	.	.	A	.	A	.	.	C	.	.	.	G	.	T	A	C	T	C	C	.	.	T	.	A	T	C	C	G	.	C	.	T	.	.
16	.	.	A	.	A	T	.	C	.	.	.	G	.	T	A	C	T	C	C	.	.	T	.	A	T	C	C	G	.	C	.	T	.	.
17	.	.	A	.	G	T	.	T	.	.	.	G	.	T	A	T	T	C	C	.	.	C	.	A	C	T	C	G	.	C	.	T	.	.
18	.	.	C	.	G	C	.	T	.	.	.	A	.	C	G	T	A	C	A	.	.	C	.	G	C	T	T	A	.	G	.	C	.	.
19	.	.	C	.	G	C	.	T	.	.	.	A	.	C	G	T	A	T	A	.	.	C	.	G	C	T	T	A	.	G	.	C	.	.
20	.	.	A	.	A	T	.	C	.	.	.	G	.	C	A	C	T	C	C	.	.	T	.	A	T	C	C	G	G	C	.	T	.	.
21	.	.	A	.	A	T	.	C	.	.	.	G	.	T	A	C	T	C	C	.	.	T	.	A	T	C	C	G	.	C	A	T	.	.
22	.	.	C	.	G	C	.	T	.	.	.	A	.	C	G	T	A	C	A	.	.	C	.	G	C	T	T	A	.	G	.	C	.	A
23	.	.	C	.	G	C	A	T	T	.	.	A	.	C	G	T	A	C	A	.	.	C	.	G	C	T	T	A	.	G	.	C	.	.
24	.	.	C	.	G	T	.	T	.	.	.	A	.	C	G	T	A	T	A	.	.	C	.	G	C	T	T	A	.	G	.	C	.	.
25	.	.	C	.	G	C	.	T	.	.	.	A	T	C	G	T	A	C	A	.	.	C	.	G	C	C	C	G	.	G	.	C	.	.
26	T	T	A	.	A	T	.	C	.	.	.	G	.	T	A	C	T	C	C	.	.	T	.	A	T	C	C	G	.	C	.	T	.	.
27	.	.	C	.	G	C	.	T	.	A	A	T	.	T	G	C	T	.	.	.	.	C	.	G	.	.	.	G	.	.	.	.	.	.
28	.	.	A	Y	A	T	.	C	.	.	.	G	.	T	A	C	T	C	C	.	.	T	.	A	T	C	C	G	.	C	.	T	M	.
29	T	T	A	.	A	T	.	C	.	.	.	G	.	.	A	C	T	C	C	.	.	T	.	A	T	C	C	G	.	C	.	T	.	.
30	.	.	C	.	G	C	.	T	.	.	.	A	.	C	G	T	A	T	C	.	.	T	.	G	T	T	T	A	.	G	.	T	.	.
	*	*	*		*	*		*				*		*	*	*	*	*	*			*	*	*	*	*	*	*		*		*		*

Genotype 1: LM38, LM30, LM 47, LM42, LM48, LM32, LM141, LM127, LM3, LM44, LM50, LM6, LM9, LM96, LM17, LM14, LM114, LM116, LM139, LM133; **Genotype 2**: LM174; **Genotype 3**: LM153; **Genotype 4**: LM72, LM54, LM136; **Genotype 5**: LM28; **Genotype 6**: LM36; **Genotype 7**: LM142; **Genotype 8**: CBS 1555, CBS 7858, CBS 608, CBS 712, CBS 397, LM92; **Genotype 9**: CBS 7894; **Genotype 10**: 6M2; **Genotype 11**: CBS 834; **Genotype 12**: LM15; **Genotype 13**: LM20, 1SC4; **Genotype 14**: NCYC 1424, CBS 5795, LM161; **Genotype 15**: CBS 5668; **Genotype 16**: VG6, LM154; **Genotype 17**: UCKM1; **Genotype 18**: CBS 4857, LM 167, LM 148, DMB1; **Genotype19**: NCYC 179; **Genotype 20**: CBS 745; **Genotype 21**: CBS 1596; **Genotype 22**: DMKU 3-1042, NBRC 1777, M41, M81, M83, VG4, VG1; IIPE453; **Genotype 23**: CBS 4354, CBS 2233; **Genotype 24**: CBS 6432; **Genotype 25**: CBS 6556, KCTC 17555; **Genotype 26**: CCT 7735, K326, M12, M123, M131, M135, M14, M166, M169, M38, M48, M68; **Genotype 27**: LM169; **Genotype 28**: CBS 2762; **Genotype 29**: FM09; **Genotype 30**: BO339.

Continued on following page

Table S2 – *Continued*

*SGA*1 Genotype	Site position 42	43	46	47	97	108	112	190	191	192	210	216	228	241	258	267	349	383	394	427	437	467	482	483	500	519	539	542	588	597	603	606	634	651	657	672	742	775	795	800	828	852	907	920
1	C	T	C	A	C	R	C	C	C	C	T	C	G	C	Y	C	A	C	C	C	A	C	C	T	C	Y	G	G	R	R	Y	C	G	Y	R	G	R	S	T	T	C	Y	G	G
2	.	.	.	.	.	.	.	.	.	.	.	.	.	.	.	.	R	.	.	.	.	.	.	.	.	.	.	.	.	.	.	.	.	.	.	.	.	.	.	.	.	.	.	.
3	.	.	.	.	.	G	.	.	.	.	.	.	.	.	.	.	.	.	.	.	.	.	.	.	.	.	.	.	.	.	.	.	.	.	.	.	.	.	.	.	.	.	.	.
4	.	.	.	.	.	.	.	.	.	.	.	.	.	.	.	.	.	.	.	.	.	.	.	.	.	C	.	.	.	.	.	.	.	.	.	.	.	.	.	.	.	.	.	.
5	.	.	.	.	.	.	.	.	.	.	.	.	.	.	.	.	.	.	.	.	.	.	.	.	.	C	.	.	.	.	.	.	.	.	.	.	.	C	.	.	.	.	.	.
6	.	.	.	.	.	.	.	.	.	.	.	.	.	.	.	.	.	.	.	.	.	.	.	.	.	C	.	.	A	G	.	.	.	?	A	.	?	C	.	.	.	T	.	T
7	.	.	.	.	.	A	.	.	.	.	.	.	.	.	C	.	.	.	.	.	.	.	.	.	.	T	.	.	G	A	C	.	.	C	G	.	G	G	.	.	.	C	.	.
8	.	.	.	.	.	G	.	.	.	.	.	.	.	.	T	.	.	.	.	.	.	.	.	.	.	T	.	.	A	G	T	.	.	T	A	.	G	C	.	.	.	T	.	.
9	.	.	.	.	.	A	.	.	.	.	.	.	.	.	C	.	.	.	.	.	.	.	.	.	.	T	.	.	G	A	C	T	.	C	G	.	G	G	.	.	.	C	.	.
10	.	.	.	.	.	A	.	.	.	.	.	.	.	.	C	.	.	.	.	.	.	.	.	A	.	T	.	.	G	A	C	.	.	C	G	A	G	G	.	.	T	C	.	.
11	.	.	.	.	.	A	.	.	.	.	.	.	C	.	C	G	.	.	.	.	.	.	.	.	.	T	.	.	G	A	C	.	.	C	G	.	G	G	.	.	.	C	.	.
12	.	.	.	.	.	G	.	.	.	.	.	.	.	.	C	.	.	.	.	.	.	.	.	.	.	C	A	.	A	G	T	.	.	T	G	.	A	G	.	.	.	C	.	.
13	.	.	.	.	.	G	.	.	.	.	.	.	.	.	C	G	.	.	.	.	.	.	.	.	.	T	.	.	G	A	C	.	.	C	G	.	G	G	.	.	.	C	.	.
14	.	.	.	.	.	G	.	.	.	.	.	.	.	.	T	.	.	.	.	.	.	.	.	.	.	C	.	.	A	G	T	.	.	T	A	.	A	C	.	.	.	T	.	.
15	.	.	.	.	.	G	.	.	.	.	.	.	.	.	T	.	.	.	.	.	.	.	.	.	.	C	.	.	A	G	T	.	.	T	A	.	A	C	.	.	.	T	.	.
16	.	.	.	.	.	G	.	.	.	.	.	.	.	.	T	.	.	.	.	.	.	.	.	.	.	C	.	.	A	G	T	.	.	T	A	.	A	C	.	.	.	T	T	.
17	.	.	.	.	.	G	.	.	.	.	.	.	.	.	T	.	.	.	.	.	G	.	.	.	.	C	.	.	A	G	T	.	.	T	A	.	A	C	.	.	.	T	.	.
18	.	.	.	.	.	G	.	.	.	.	.	.	.	.	T	.	.	.	.	.	.	.	.	.	Y	C	.	.	A	G	T	.	.	T	A	.	A	C	.	.	.	T	.	.
19	.	.	.	.	.	A	.	.	.	.	.	.	S	.	C	S	.	.	.	.	.	.	.	.	.	T	.	.	G	A	C	.	.	C	G	.	G	G	.	.	.	C	.	.
20	.	.	.	.	.	G	.	.	.	.	.	.	.	.	T	.	.	.	.	.	R	.	.	.	.	C	.	.	A	G	T	.	.	T	A	.	A	C	.	.	.	T	.	.
21	.	.	.	.	.	G	.	.	.	.	.	M	.	.	T	.	.	.	.	.	.	.	.	.	.	C	.	.	A	G	T	.	.	T	A	.	A	C	.	.	.	T	.	.
22	.	.	.	.	M	.	M	.	.	.	.	.	.	.	.	.	.	.	.	.	.	.	.	.	.	C	.	.	.	.	.	.	R	.	.	.	G	.	.	.	.	.	.	.
23	.	.	.	.	M	.	M	.	.	.	.	.	.	.	.	.	.	M	M	.	.	M	M	.	.	.	.	R	.	.	.	.	R	.	.	.	G	.	.	.	.	.	.	.
24	.	.	.	.	M	G	M	M	M	M	W	.	.	M	T	M	.	M	M	M	.	M	M	.	.	C	.	.	A	G	T	.	A	T	A	.	G	G	.	.	.	C	.	.
25	A	C	T	C	.	.	.	.	.	.	.	.	.	.	.	.	.	.	.	.	.	.	.	.	.	.	.	.	.	.	.	.	.	.	.	.	.	.	.	.	.	.	.	.
26	A	C	T	C	.	A	.	.	.	.	.	.	C	.	C	G	.	.	.	.	.	.	.	.	.	T	.	.	G	A	C	.	.	C	G	.	G	A	A	G	.	T	.	.
	*	*	*	*	*	*	*						*		*	*		*	*		*	*	*			*			*	*	*		*	*	*		*	*				*		

Genotype 1: CBS 1596, CBS 7894, CBS 397, CBS 712, CBS 608, CBS 7858, CBS 1555, LM54, LM72, LM92, LM36, LM32, LM30, LM141, LM127, LM38, LM28, LM114, LM139, LM48; **Genotype 2**: LM6, VG6; **Genotype 3**: LM14; **Genotype 4**: M12, M48, M68, M81, M123, M131, M135, 6M2, K326, M41, M38, M83, M166, M169, CBS 834; **Genotype 5**: M14; **Genotype 6**: UCKM1; **Genotype 7**: NCYC 179, CBS 6432; **Genotype 8**: BO339; **Genotype 9**: CBS 4857; **Genotype 10**: DMKU 3-1042; **Genotype 11**: CBS 4354, CBS 2233, LM167, NBRC 1777, VG4, CBS 745, VG1, IIPE453; **Genotype 12**: DMB1; **Genotype 13**: CBS 6556, KCTC 17555; **Genotype 14**: CBS 2762, CBS 5668, NCYC 1424, CBS 5795, LM50, LM96, LM9, LM3; **Genotype 15**: LM116, LM133, LM136, LM44, 1SC4, FM09, CCT 7735; **Genotype 16**: LM 42; **Genotype 17**: LM15; **Genotype 18**: LM47; **Genotype 19**: LM20; **Genotype 20**: LM161, LM154; **Genotype 21**: LM153; **Genotype 22**: LM174; **Genotype 23**: LM17; **Genotype 24**: LM169; **Genotype 25**: LM142; **Genotype 26**: LM148

Continued on following page

Table S2 – *Continued*

GSY2	Site position																																															
Genotype	20	27	30	36	41	90	98	104	114	115	116	119	120	132	150	152	156	168	182	210	211	216	256	257	364	384	438	442	459	511	537	598	612	615	642	678	684	704	719	720	723	734	736	744	817	828	856	858	873
1	C	C	A	C	A	Y	G	C	M	C	T	C	C	C	Y	C	G	G	G	Y	C	C	C	C	G	C	Y	G	C	T	C	A	R	R	G	R	M	A	C	A	T	A	R	Y	C	C	G	T	Y
2	.	.	.	.	.	.	.	.	.	.	.	.	.	.	.	.	.	.	.	.	.	.	.	.	.	.	.	.	.	.	.	.	.	.	.	.	.	.	.	.	.	.	.	.	.	.	A	.	.
3	.	.	.	.	.	.	.	.	.	.	.	.	.	.	.	.	.	.	.	.	.	.	.	.	.	.	.	.	.	.	.	.	.	.	.	.	.	.	.	.	.	S	.	.	.	.	.	.	.
4	.	.	.	.	.	.	.	.	.	.	.	.	.	.	.	.	.	.	.	.	.	.	.	.	.	.	.	.	.	.	.	.	.	.	R	.	.	.	.	.	.	.	.	.	.	.	A	.	.
5	.	.	.	.	.	.	.	.	.	.	.	.	.	.	.	.	.	.	.	C	.	.	.	.	.	.	.	.	.	.	.	.	.	.	.	.	.	.	.	.	.	.	.	.	.	.	.	.	.
6	.	.	.	.	.	.	.	.	.	.	.	.	.	.	C	.	.	.	.	.	.	.	.	.	.	.	.	.	.	.	.	.	.	.	.	.	.	.	.	.	.	.	.	.	.	.	.	.	.
7	.	.	.	.	.	.	.	.	.	.	.	.	.	.	C	T	.	.	.	T	.	.	.	.	.	.	T	.	.	.	.	.	A	A	.	A	A	.	.	.	.	.	A	C	.	.	.	.	C
8	.	.	.	.	.	.	.	.	A	.	.	.	.	.	T	.	.	.	.	.	.	.	.	.	.	.	C	R		.	.	.	G	G	.	G	C	.	.	.	.	.	G	T	.	.	.	.	C
9	.	.	.	.	.	.	.	.	A	.	.	.	.	.	T	.	.	.	.	T	Y	.	.	.	.	.	C	.	.	.	.	.	G	G	.	G	C	.	.	.	.	.	G	T	.	.	.	.	.
10	.	.	.	.	.	.	.	.	C	.	.	.	.	.	C	.	.	.	.	.	.	.	.	.	.	.	.	.	.	.	.	.	.	.	.	.	.	.	.	.	.	.	.	.	.	.	.	G	.
11	.	.	.	.	.	C	.	.	.	.	.	.	.	.	.	.	.	.	.	.	.	.	.	.	.	.	.	.	.	.	.	.	.	.	.	.	.	.	G	C	G	.	.	T	.	.	.	Y	.
12	.	.	.	.	.	T	.	.	.	.	.	.	.	.	C	.	.	.	.	T	.	.	.	.	.	.	T	.	.	.	.	.	A	A	.	A	A	.	.	.	.	.	A	C	.	.	.	.	.
13	.	.	.	.	.	C	.	.	A	.	.	.	.	.	C	.	.	.	.	.	.	.	.	.	K	.	.	.	.	.	.	.	.	.	.	.	.	.	.	.	.	.	.	.	.	.	.	G	T
14	.	.	.	.	.	C	.	.	A	.	.	.	.	.	T	.	.	.	.	.	Y	.	.	.	.	.	C	.	.	.	.	.	G	G	.	G	C	.	.	.	.	.	G	T	.	.	.	.	.
15	.	.	.	.	.	C	.	.	A	.	.	.	.	.	T	.	.	.	.	.	Y	.	.	.	.	.	C	.	.	.	.	.	G	G	.	G	C	.	.	.	.	.	G	T	Y	.	.	.	C
16	.	.	.	.	.	C	.	.	A	.	.	.	.	.	T	.	.	.	.	C	.	.	.	.	.	.	C	.	.	.	.	.	.	.	.	.	.	.	.	.	.	.	G	.	.	.	.	.	C
17	.	.	.	.	.	C	.	.	A	.	.	.	.	.	T	.	.	.	.	C	.	.	.	.	.	.	C	.	.	.	.	.	.	.	.	G	C	.	.	.	.	.	G	T	.	.	.	.	.
18	.	.	.	.	.	C	.	.	A	.	.	.	.	.	T	.	.	.	.	C	.	.	.	.	.	.	C	.	.	.	.	.	G	G	.	G	C	.	.	.	.	.	G	T	.	.	.	.	T
19	.	.	.	.	.	C	.	.	A	.	.	.	.	.	T	.	.	.	.	C	.	.	.	.	.	.	C	.	.	.	.	.	G	G	.	G	C	.	.	.	.	.	G	T	.	.	.	G	C
20	.	.	.	.	.	C	.	.	A	.	.	.	.	.	T	.	.	.	.	C	.	.	.	.	.	.	C	.	.	.	.	.	G	G	.	G	C	.	.	.	.	.	G	T	.	.	A	.	T
21	.	.	.	.	.	T	.	.	C	.	.	.	.	.	C	.	.	.	.	T	.	.	.	.	.	.	C	.	.	.	.	.	A	A	.	G	C	.	.	.	.	.	A	C	.	T	.	.	T
22	.	.	.	.	.	T	.	.	C	.	.	.	.	.	C	.	.	.	.	T	.	.	.	.	.	.	T	.	.	.	.	.	A	A	.	A	A	.	.	.	.	.	A	C	.	.	.	.	T
23	.	.	.	.	.	T	.	.	C	.	.	.	.	.	C	.	.	.	.	T	.	.	.	.	.	.	T	.	.	.	.	.	A	A	.	G	A	.	.	.	.	.	A	C	.	.	.	.	T
24	.	.	.	.	.	T	C	.	C	.	.	.	.	.	C	.	.	.	.	T	.	.	.	.	.	.	T	.	.	.	.	.	A	A	.	A	A	.	.	.	.	.	A	C	.	.	.	G	T
25	.	.	.	.	.	C	.	.	A	.	.	.	.	.	T	.	.	.	.	T	.	.	.	.	.	T	C	.	.	.	.	.	G	G	.	G	C	.	.	.	.	.	G	T	.	.	.	.	C
26	.	.	.	.	.	T	.	.	A	.	.	.	.	.	T	.	.	A	.	T	.	.	.	.	.	.	C	.	A		.	.	G	G	.	G	C	.	.	.	.	.	G	T	.	.	.	.	C
27	.	.	.	.	.	T	.	.	C	.	.	.	.	.	C	.	.	.	.	T	.	.	.	.	K	.	T	.	.	.	.	.	A	A	.	A	A	.	.	.	.	.	A	C	.	.	.	G	T
28	.	.	.	.	.	C	.	.	A	.	.	.	.	.	T	.	.	.	.	C	.	.	.	.	K	.	C	.	.	.	.	.	G	G	.	G	C	.	.	.	.	.	G	T	.	.	.	G	T
29	.	.	.	.	.	Y	.	.	A	.	.	.	.	.	T	.	.	.	.	.	.	.	.	.	.	.	C	R		.	.	.	G	G	.	G	C	.	.	.	.	.	G	T	.	.	.	.	C
30	C	.	.	.	.	C	.	.	A	.	.	.	.	.	T	C	.	.	.	T	.	.	.	.	.	.	C	.	.	.	.	.	.	G	.	G	C	.	.	.	.	.	G	T	.	.	.	.	C
31	.	.	.	.	.	T	.	.	C	M	.	.	.	M	C	.	.	.	.	T	.	S	.	.	.	.	T	.	.	W	M	W	A	A	.	A	A	G	.	.	.	.	G	T	.	.	.	.	C
32	.	.	.	.	.	C	.	M	A	.	W	M	M	M	T	.	R	.	R	.	Y	.	M	M	.	.	C	.	.	.	.	.	G	G	.	G	C	.	.	.	.	.	G	T	.	.	.	G	T
33	G	A	C	G	G	C	.	.	C	.	.	.	.	.	.	.	.	.	.	.	.	.	.	.	.	.	.	.	.	.	.	.	A	A	.	A	A	.	.	.	.	.	.	C	.	.	.	.	
34	.	.	.	.	.	C	.	.	A	.	.	.	.	.	T	.	.	.	.	T	.	.	.	.	.	.	T	.	.	.	.	.	G	G	.	G	C	.	.	.	.	.	G	T	.	.	.	.	T
	*					*			*					*	*	*				*	*				*		*		*				*	*		*	*						*	*			*	*	*

Genotype 1: CBS 834, CBS 1596, CBS 397, CBS 712, CBS 608, NCYC 179, CBS 6432, CBS 7858, CBS 1555, LM127, LM42, LM44, LM47, LM50, LM54, LM72, LM9, LM96, LM38, LM36, LM30, LM3, LM141, LM133, LM139, LM14; **Genotype 2**: LM32; **Genotype 3**: 6M2; **Genotype 4**: LM6; **Genotype 5**: LM48; **Genotype 6**: LM136; **Genotype 7**: LM116; **Genotype 8**: CBS 2762; **Genotype 9**: NCYC 1424, CBS 5795; **Genotype 10**: LM17; **Genotype 11**: LM153; **Genotype 12**: 1SC4; **Genotype 13**: LM169; **Genotype 14**: LM161; **Genotype 15**: LM15; **Genotype 16**: K326; **Genotype 17**: VG6, LM142, M68, M81, M12, M48, M14, M123, M131, M38, M41, M83, M135, M166, M169; **Genotype 18**: LM114; **Genotype 19**: LM174; **Genotype 20**: LM92; **Genotype 21**: CBS 6556, KCTC 17555; **Genotype 22**: CBS 7894, VG1, DMKU3-1042, DMB1, NBRC 1777, VG4, IIPE453; **Genotype 23**: CBS 4354, CBS 4857, CBS 2233; **Genotype 24**: LM20; **Genotype 25**: CBS 5668; **Genotype 26**: CBS 745; **Genotype 27**: LM167; **Genotype 28**: LM28; **Genotype 29**: FM09; **Genotype 30**: CCT7735; **Genotype 31**: LM148; **Genotype 32**: LM154; **Genotype 33**: UCKM1; **Genotype 34**: BO339
